# Adenoma of the Breast in a Dog

**Published:** 1883-01

**Authors:** Frank V. Walton

**Affiliations:** Surgeon to Columbia Veterinary College Hospital


### II.—ADENOMA OF THE BREAST IN A DOG.

REPORTED BY FRANK V. WALTON, D.V.S.
*Surgeon to Columbia Veterinary College Hospital.*

The animal afflicted was a large bitch about six years old. The new growth involved the anterior half of the right breast, and hung down as an irregular nodular mass. The growth was hard and of about one year's duration. The animal was in good flesh and apparently quite well in general health. Upon the same side in the inguinal region there was another large and pendulous tumor of several years standing. This tumor, however, was perfectly smooth, soft and doughy, and could be greatly diminished in size. This tumor was diagnosticated, as an inguinal hernia.

The tumor of the breast was removed at one of the surgical clinics of the Columbia Veterinary College by Prof. Porter. The ordinary elliptical incisions commonly resorted to in the amputation of the breast of the human female were made. But instead of making them transversely to the trunk they were made longitudinally. This large, irregular and nodulated mass was dissected off from the chest wall, leaving a sufficient quantity of integument to close the wound. Several small arteries were cut, but torsion was all that was required to arrest the hemorrhage. The capillary oozing readily ceased upon applying cold water and the exposure of the lips to the air. The lips of the wound were brought together by fine sutures and the edges of the skin closely and evenly approximated so that as perfect homogeneity of tissue as possible was maintained. The dog speedily recovered from the influence of the ether and apparently did not suffer at all from shock. The following day the animal was carefully taken home. In about one week she was again brought to the College, where it was found that nearly the whole length of the incision had united by first intention. At one point, not more than one centimetre or one-half inch in length, it had not united, but was rapidly closing by granulating from the bottom. The length of the original wound when sewed up was about thirteen centimetres, or five inches. One week later the wound had completely healed and the result was a perfect union by first intention; the scar was very slight and probably in a few months will be wholly imperceptible.

*Microscopic Report of Tumor.*—The tumor weighed 7,464 grains, or one and one-half pounds. It was distinctly lobulated, as is commonly observed in this orm of growth. Sections made through these nodules showed an increase in size of the mammary gland ducts with an apparent hypertrophy of the gland substance. The epithelium of the tubes was also enlarged and showed degenerative changes. There was also an increase in the interstitial tissue, and at points it looked as if alveolar spaces were being formed, filled with elements somewhat resembling those commonly seen in scirrhous carcinoma of the breast. Yet no positive evidence of scirrhous was detected, and the growth may be classed as an adenoma.

[This is an exceedingly interesting case, surgically and pathologically. In its surgical aspect it is of great interest in showing what perfect results can be obtained by care and close attention to the rules laid down for treating incised wounds, namely, cleanliness, arrest of hemorrhage, removal of all foreign bodies, as dirt and hair and a perfect approximation of surfaces. There was one favorable circumstance in this case, and that was, that there was but little hair over the tumor and at the line of incision. Pathologically, this case is interesting in being a true adenoma, a tumor which is not often met with in human pathology.

It is also of intesest in showing a close resmblance to a scirrhous. Had true scirrhous carcinomatous structure been met with, it would have been classed as an adeno-carcinoma. As all the readers of this Journal may not be familiar with the meaning of the terms "adenoma" and "carcinoma" we would say that adenoma, strictly speaking, is a hypertrophy and new development of the conglomerate gland structure as the mammary gland, parotid and salivary glands and pancreas, etc.

A scirrhous carcinoma is one in which broad bands of tendon like tissue form open spaces, and these spaces are packed full of large, irregular shaped epithelial elements, and in these spaces there are no intercellular elements, and in this respect the carcinoma or cancer differs from the sarcoma. In the sarcoma there is always some intercellular substance.—ED.]

